# Keystone Taxa *Lactiplantibacillus* and *Lacticaseibacillus* Directly Improve the Ensiling Performance and Microflora Profile in Co-Ensiling Cabbage Byproduct and Rice Straw

**DOI:** 10.3390/microorganisms9051099

**Published:** 2021-05-20

**Authors:** Guilin Du, Guilong Zhang, Jiping Shi, Jingxian Zhang, Zhiguo Ma, Xiangcen Liu, Chenyang Yuan, Xiang Li, Baoguo Zhang

**Affiliations:** 1 Lab of Biorefinery, Shanghai Advanced Research Institute, Chinese Academy of Sciences, No. 99 Haike Road, Pudong, Shanghai 201210, China; dugl1@shanghaitech.edu.cn (G.D.); shijp@sari.ac.cn (J.S.); zhangjingxian@sari.ac.cn (J.Z.); mazhiguo2018@sari.ac.cn (Z.M.); liuxiangcen2019@sari.ac.cn (X.L.); yuanchy@shanghaitech.edu.cn (C.Y.); 2 University of Chinese Academy of Sciences, Beijing 100049, China; 3 School of Life Science and Technology, Shanghai Tech University, Shanghai 201210, China; 4 Agro-Environmental Protection Institute, Ministry of Agriculture and Rural Affairs, Tianjin 300191, China; zhangguilong@caas.cn

**Keywords:** vegetable waste, silage, *Lactiplantibacillus plantarum* inoculation, microbial community dynamics, microbial phenotypes, potential pathogen, path analysis

## Abstract

Ensiling has been widely applied to cope with agricultural solid waste to achieve organic waste valorization and relieve environmental pressure and feedstuff shortage. In this study, co-ensiling of cabbage leaf byproduct and rice straw was performed with inoculation of *Lactiplantibacillus* *plantarum* (LP) to investigate the effects of inoculation on ensiling performance and microflora profiles. Compared to the control, LP inoculation preserved more dry matter (DM) content (283.4 versus 270.9 g·kg^−1^ fresh matter (FM) on day 30), increased lactic acid (LA) content (52.1 versus 35.8 g·kg^−1^ dry matter on day 15), decreased pH (3.55 versus 3.79 on day 15), and caused accumulation of acetic acid (AA), butyric acid (BA), and ammonia. The investigation showed that LP inoculation modified microflora composition, especially resisting potential pathogens and enriching more lactic acid bacteria (LAB) (*p* < 0.05). Moreover, *Lactiplantibacillus* and *Lacticaseibacillus* were identified as the keystone taxa that influenced physicochemical properties and interactions in microflora. They were also the main functional species that directly restrained undesirable microorganisms (*p* < 0.05), rather than indirectly working via metabolite inhibition and substrate competition (*p* > 0.05). The results of this present study improve the understanding of the underlying effect of LP inoculation on improving silage quality and facilitate the bio-transformation of cabbage byproduct and rice straw as animal feed.

## 1. Introduction

With population and living standard rapidly rising, a series of social problems have been more prominent, including a shortage of food, food–feed competition for arable land, and a vast amount of waste from agriculture and the food processing industry [1,2]. Vegetable wastes (e.g., leaves of cabbage, cauliflower, and amaranth) and rice straw are two common types of waste generated from consumption of leaf vegetables and rice as important food sources. According to the statistics, 800 million tons of vegetable waste and 900 million tons of crop straw are annually generated from harvesting to sale in China [2,3,4]. Generally, vegetable wastes are highly moist and perishable, while rice straw is dry and degrades slowly. This would pose huge challenges to the downstream processing of individual waste. As previously reported, a considerable quantity of vegetable wastes (around 240 million) and most crop straws (around 180 million) are irrationally disposed of in China, for instance, directly dumping to landfill and burning in a field, which has caused atmospheric and soil pollution, greenhouse gas emission, and pathogen transmission [2,4,5]. The current situation has created an urgent need to develop more sustainable strategies for the effective processing of quickly produced organic wastes.

Among the currently available techniques for organic waste treatment, ensiling was reported to be highly efficient, easy to operate, and environmentally friendly. The obtained silage products can serve as low-cost animal feeds which facilitate the development of the sustainable livestock industry. Ensiling is an anaerobic fermentation process in which the epiphytic microorganisms (mainly LABs) metabolize water-soluble carbohydrates (WSC) to organic acids (e.g., LA, AA, BA) [2]. However, the presence of some pathogens (e.g., *Escherichia coli*, *Fusarium verticillioides*), spore-forming bacteria (e.g., *Clostridium botulinum*), and heterofermentative LABs (e.g., *Lentilactobacillus buchneri*) may result in the accumulation of various microorganic toxins and inefficient LA fermentation, impairing the quality of silage products [6,7,8]. The successful ensiling process would rely on the appropriate composition of feedstock (e.g., contents of moisture and WSC) as well as the existing microbial community. For example, Mu et al. performed co-ensiling of amaranth and rice straw, which achieved good preservation of high-moisture amaranth [9]. In the study of Wang et al. [10], *Neolamarckia cadamba* leaves were co-ensiled with high moisture alfalfa and stylo silage, which improved the quality of silage characterized with lower pH and ammonia-N content, and higher LA and true protein content. Ren et al. also reported that co-ensiling was a feasible strategy to produce high-value bioproducts from maize straw and cabbage waste [4].

Research efforts have also been put to investigate the inoculation of exogenous probiotics to modify the configuration of the microbial community and improve the desired functions. Homofermentative *Lpb. plantarum* (LP), *Lacticaseibacillus casei*, *Lcb. rhamnosus*, and heterofermentative *Lentilactobacillus buchneri* have been successfully applied in ensiling of different feedstocks, which could improve the ensiling quality due to the inhibition of the growth and metabolism of the microorganisms characterized by pathogens and deteriorations [2,6,11,12,13]. The external inoculation improved silage quality and changed the microbial community composition, mostly illustrated by relative abundance (RA) of different species. Recent studies have increasingly reported that it is keystone taxa, rather than the most abundant ones, mainly explaining the dynamics of ecological microflora and environmental properties [14,15]. Therefore, it is essential and meaningful to investigate how keystone taxa occur together in niches and play an important role in improving silage quality. Furthermore, the change of functional diversity in response to LP inoculation and the underlying effect of improvement by external additives are of great importance for understanding the ensiling process, and still remain unclear.

In this study, one kind of common vegetable waste, cabbage leaf byproduct, was co-ensiled with rice straw as a sustainable strategy for waste processing. Exogenous LP was inoculated to investigate its effects along the entire process of 30 days. The ensiling profiles, dynamic of microbial community and characteristics (phenotypes and functional guilds), and the identification and transition of keystone taxa were thoroughly analyzed. The underlying intricate relationships of how LP inoculation influenced the fermentation characteristics and microflora were comprehensively investigated.

## 2. Materials and Methods

### 2.1. Preparation of Materials and Additives

Cabbage (*Brassica oleracea* L.) leaf byproducts were collected from Beicai Agricultural Products Wholesale Market (Shanghai, China). Rice straw was purchased from the Changyuan Straw Processing Plant (Anhui, China). Both cabbage leaves and rice straws were shredded to a length of 1–2 cm. The compositions of raw materials are shown in Table 1, and were measured following the procedure in Section 2.4. It was previously proved that homolactic fermentation and facultative anaerobic LP (CGMCC NO.19862) could improve the cabbage byproduct fermented feed; they were stored at –80 °C in our laboratory using strain preservation tubes with MRS medium containing 35% (*v*/*v*) glycerol [16]. The LP inoculation was prepared as Ren et al. reported and the final cell concentration of inoculum was 3.3 × 10^8^ CFU·mL^−1^ [2].

### 2.2. Silage Set-Up and Sampling

To ensure suitable moisture and enough energy for FM, the formula of mixed raw material was 180 g cabbage leaf, 40 g rice straw, and 40 g corn flour per bag. To inoculate more equably, 5 mL LP inoculation, as in Section 2.1., was diluted with 100 mL 0.8% sterile NaCl (Sinopharm, Shanghai, China) solution and shaken well. Diluted LP inoculation of 5 mL was added per bag for the treatment group (LPGP, LP content: 5.9 × 10^5^ CFU·g^−1^ FM), while equal-volume sterile NaCl solution was added in raw material to replace the inoculation and served as the control group (CKGP, without inoculation). The mixed raw material was packed into PET plastic bags (23 cm × 30 cm) and sealed with a vacuum sealer (Blueberry 320X, Shanghai Inuo Packaging Materials Co., Ltd., Shanghai, China). Silage bags were incubated at 30 °C for 3, 7, 15, and 30 days. A total of 30 silage bags was divided into 2 treatments and 3 replicates for 5 sampling times (2 × 3 × 5 = 30). The silage sample of 20 g was taken from each treatment for analysis of microbial community and physicochemical parameters.

### 2.3. Analytical Sample Preparation

The first lixivium was prepared by each mixing ensiling sample of around 20 g with 180 mL sterile NaCl solution (0.8% *w*/*v*) in a 500 mL conical flask. The mixture was agitated at 30 °C for 2 h using a rotary shaker at 200× rpm and filtered through four layers of sterile medical gauze under negative pressure. The filtrate was collected, centrifuged at 4 °C for 20 min at 10,000× *g* to obtain the sediment for DNA extraction and sequencing. The acquisition of the second lixivium was similar to the first one except that NaCl solution was substituted with sterile water. The supernatant was gathered after centrifugation and used for analysis of physicochemical parameters [17]. The silage sample was oven-dried at 65 °C until the weight was stable, and then ground and sieved through a 1 mm screen for subsequent nutritional composition analyses.

### 2.4. Analyses of the Physicochemical Properties

The physicochemical parameters included the principal nutritional composition (the content of crude protein (CP), crude fat (CF), neutral detergent fiber (NDF), acid detergent fiber (ADF)) and fermentation characteristics (DM, WSC, pH, organic acids (FA, LA, AA, PA, and BA), ethanol, and ammonia nitrogen/total nitrogen content).

The silage samples were oven-dried at 65 °C for 72 h to determine the DM content [2]. As in Section 2.3., 0.2 g dry sample was digested under 430 °C with H_2_SO_4_ (18.4 mol/L; Sinopharm, Shanghai, China) for 1 h and then the CP content was determined using a Kjeldahl nitrogen analyzer [10]. CF content was determined as the DM loss via Soxhlet extraction (B-811, BUCHI, Flawil, Switzerland) using ethanol as solvent and oven-dried at 65 °C for 72 h. The defatted sample was subsequently used to measure ADF and NDF contents using an automatic Fibretherm (C. Gerhardt, Königswinter, Germany) per the manufacturer’s instructions [2,18]. For fermentation characteristics, the contents of organic acids and ethanol were detected using a high-performance liquid chromatography system (20AVP, Shimadzu Corp., Kyoto, Japan) with a RID-10A refractive index detector (AminexHPX-87H column (300 × 7.8 mm), Bio-Rad, Hercules, CA, USA). The oven temperature was set at 65 °C and 0.005 mol/L H_2_SO_4_ solution was used as mobile phase at a velocity of 0.8 mL/min. The retention time of LA, FA, AA, PA, BA, and ethanol was 9.9, 10.3, 11.3, 13.0, 14.7, and 16.2 min, respectively. All the standard reagents of organic acids and ethanol were purchased from Sigma-Aldrich Co., Ltd. (Burlington, Vermont, USA). Total organic acid (TOA) was calculated using the summation of LA, FA, AA, PA, BA. The silage quality was evaluated using Flieg’s score based on the organic acid ingredient (score ≥ 81, very good; 61 ≤ score < 81, good; 41 ≤ score < 61, medium; 21 ≤ score < 41, bad; score < 21, very bad) [4]. Ammonia-N was quantified using Nessler’s reagent (Hach, Loveland, CO, USA) and a spectrophotometer (DR2800; Hach, USA) at 420 nm. The second lixivium, as in Section 2.3, was used to determine pH with a digital pH meter (PB-10, Sartorius, Arvada, CO, USA) per the manufacturer’s instructions. Around 20 mg dry sample, as in Section 2.3 was parcelled using tin paper and used to detect total nitrogen using an elemental analyzer (PerkinElmer SERIES ll 2400, Waltham, MA, USA). The WSC was extracted from a fresh sample by boiling water and quantified via a microplate reader (BioTek, Winooski, VT, USA) at 630 nm using the anthrone method [2,19].

### 2.5. DNA Extraction and MiSeq Sequencing

For the microbial community, total DNA was extracted using an E.Z.N.A_._^®^ soil DNA Kit (Omega Bio-Tek, Norcross, GA, USA) per the manufacturer’s instructions. DNA purity and concentration were measured using a NanoDrop 2000 UV-vis spectrophotometer (Thermo Scientific, Wilmington, NC, USA). All DNA samples were stored at −80 °C until required. PCR amplification of prokaryotic 16S rDNA and eukaryotic internal transcribed spacer (ITS) regions were performed as described previously [10]. The V3–V4 hypervariable regions from 16S rRNA were amplified barcoded fusion primers 338F (ACTCCTACGGGAGGCAGCAG) and 806R (GGACTACHVGGGTWTCTAAT). The amplification of the ITS region from ITS rRNA used ITS1F (CTTGGTCATTTAGAGGAAGTAA) and ITS2R (CTTGGTCATTTAGAGGAAGTAA). The DNA quality was confirmed by 1.5% agarose gel electrophoresis. PCR products were sent to Majorbio Bio-pharm Technology Co., Ltd. (Shanghai, China) for further purification, extraction, and sequencing, as described previously [20].

### 2.6. Bioinformatics Analyses

The raw Illumina fastq files were demultiplexed, quality filtered, and analyzed using QIIME (v. 1.9.1 http://qiime.org/, accessed on 23 October 2020). The quality-filtered sequences were clustered into operational taxonomic units (OTUs) with a 97% similarity threshold using UPARSE (v. 7.1 http://drive5.com/uparse/, accessed on 23 October 2020) and chimeric sequences were removed using UCHIME. The taxonomy of each 16S and ITS rDNA sequence was annotated by alignment with Silva 138 (https://www.arb-silva.de/, accessed on 23 October 2020) using the RDP Classifier (v. 2.2; http://sourceforge.net/projects/rdp-classifier/, accessed on 23 October 2020) with a confidence cut-off of 70%. The MiSeq sequencing data were analyzed on the free Majorbio Cloud Platform (https://www.majorbio.com, accessed on 23 October 2020). Alpha diversity indexes (Coverage, Shannon, Chao1, Simpson, Ace) were calculated in QIIME at the OTU level. The effect of LP additives on the microflora succession was assessed using principal coordinates analysis (PCoA) associated with the Adonis test on the OTU level (999 random permutations, Bray–Curtis dissimilarity). The significantly different taxa in the control and treatment groups were identified using Wilcoxon rank-sum test (confidence interval method, *p* < 0.05). The bacterial phenotypes were manually annotated using the DacDive database (the Bacterial Diversity Metadatabase; https://bacdive.dsmz.de/, accessed on 23 October 2020) and Bergey’s Manual of Systematic Bacteriology, and only taxa of RA > 5% were considered [21,22]. The fungal trophic modes and ecological guilds were annotated using FUNGuild (http://www.funguild.org/, accessed on 23 October 2020) [23].

### 2.7. Statistical Analysis

Fermentation characteristics and nutrition content were analyzed using two-way ANOVA for a 2 × 5 (2 treatments × 5 sampling times) full factorial experimental design with three replicates (IBM SPSS 26.0, New York City, NY, USA). The significant differences between the two groups were determined by the Tukey test (α = 0.05 and *P*_critical_ = 0.05). Correlations among dominant genera (RA > 1%) were evaluated using Spearman correlation analysis. Only important interrelationships were considered (|Spearman coefficient| > 0.7, *p* < 0.01) in the co-occurrence network and visualized using Gephi 0.9.2. The relationships among the physicochemical indexes and dominant taxa (the top 5 bacterial and fungal genera) in these two groups were evaluated using RDA (Canoco 5.0, Ithaca, NY, USA). The direct and indirect effects among the functional microflora, inhibited microflora, microbial metabolite, and fermentation substrate were evaluated using partial least squares path modeling (PLS-PM) (SmartPLS 3.0, Boenningstedt, Schleswig-Holstein, Germany) [24]. Functional and inhibited microflora were identified using factor analysis (IBM SPSS 26.0, New York City, NY, USA).

## 3. Results

### 3.1. The Physicochemical Properties of Silage

Upon the completion of ensiling process, physicochemical properties including fermentation characteristics and nutritional composition are shown in Table 2 and Table 3, respectively. The pH value and content of DM and WSC significantly declined as the fermentation time elongated while the content of LA, AA, and ethanol significantly increased in all groups. The pH was nearly neutral at the initial phase and slowly decreased to below 4.0 on day 15. Interestingly, the LP inoculation resulted in a lower pH value (3.55~5.87 in LPGP vs. 3.79~5.93 in CKGP, *p* < 0.001). In particular, pH rapidly decreased to below 4.0 on day 3 in LPGP. The DM content rapidly declined from 309.5 to 270.9 g·kg^−1^ FM in CKGP. Furthermore, the LP inoculation preserved more DM content (283.4~310.3 g·kg^−1^ FM in LPGP vs. 270.9~309.5 g·kg^−1^ FM in CKGP, *p* < 0.001). The WSC content decreased rapidly on day 3 and kept stable after day 7 in CKGP. The LPGP presented higher WSC content at day 3 than CKGP (*p* < 0.05), while there was no significant difference after day 7 (*p* > 0.05). The organic acid and ethanol content increased first to the maximum (35.8 g·kg^−1^ DM) and then declined to 22.3 g·kg^−1^ DM at the end in CKGP. Regarding LPGP, the LP inoculation resulted in higher LA content (0~52.1 g·kg^−1^ DM in LPGP vs. 0~35.8 g·kg^−1^ DM in CKGP, *p* < 0.001) of silage samples, while there were lower contents of AA, and BA (*p* < 0.001), which led to higher LA/TOA and lower AA/TOA, BA/TOA (Table 4). Regarding the silage quality based on the organic acid ingredient, only the samples on day 15 were evaluated as “good” (Flieg’s score > 60) in CKGP and other scores were evaluated below 60. However, the LP inoculation improved the silage quality (very good; Flieg’s score ≥ 81) during the overall fermentation process. Furthermore, LP inoculation played no significant role in ethanol production. Ammonia-N significantly accumulated in CKGP (*p* < 0.05; 6.4~11.9% during ensiling), and the LP inoculation significantly decreased ammonia-N content during the whole process (*p* < 0.05, below 7% during ensiling).

For nutritional components, the CF content decreased while the contents of ADF and NDF increased in both groups (Table 3). Among the various nutritional components, lignocellulose was less preferred and left, leading to an increasing mass ratio in DM in both groups. Moreover, the LP inoculation played no significant role in the accumulation of CF and CP (*p* > 0.05) but significantly decreased the content of ADF and NDF (*p* < 0.05) on day 3 and day 15. The ADF content also significantly decreased at the mature phase (day 30, *p* < 0.05).

### 3.2. Silage Bacterial and Fungal Composition

Based on 16S and ITS rDNA sequencing, the coverage index of all samples was above 0.99 (Table 5), which indicated the DNA sequencing results were representative of the microbial community. The richness was evaluated using Chao1 and Ace indexes while the diversity was done by Shannon and Simpson indexes. The Shannon index of the bacterial community significantly declined, and the Simpson index of the bacterial community significantly increased in LPGP compared with that in CKGP (*p* < 0.05); moreover, there was no significant difference between Shannon and Simpson indexes of fungi, Chao1 and Ace indexes of bacteria and fungi (*p* > 0.05).

**Figure 1:**
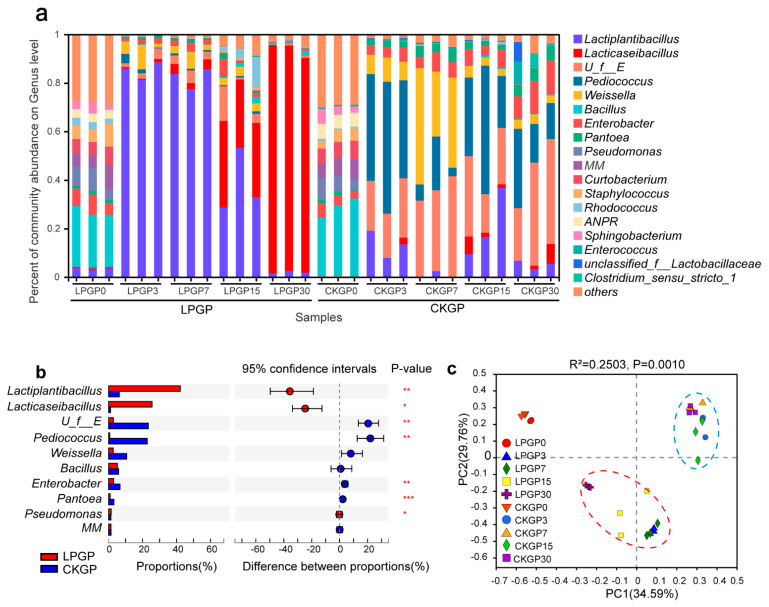
Effect of Lactiplantibacillus *plantarum* additive on the bacterial community dynamics of cabbage and rice straw silage. (**a**) The relative abundance of bacterial taxa at the genus level; taxa with <1% of reads were combined as “others”. (**b**) Wilcoxon rank-sum analysis of significant bacterial genera between silages without and with inoculation; ***, **, and * represent *p* < 0.001, *p* < 0.01, and *p* < 0.05, respectively. (**c**) Principal coordinate analysis was based on bacterial OTU level. CKGP: The control group; LPGP: The inoculation group. The numbers following the CKGP and LPGP indicate the sampling time (day). *U_f__E*, *unclassified_f__Enterobacteriaceae*; *MM*, *Methylobacterium-Methylorubrum*; *ANPR*, *Allorhizobium-Neorhizobium-Pararhizobium-Rhizobium*.

Fermentation time and LP inoculation significantly influenced the microbial community succession throughout anaerobic ensiling (*p* < 0.05, Figure 1, Figures S1a and S2a). For raw materials, Firmicutes was observed as the most dominant phylum (39.7%) followed by Proteobacteria (38.8%) and Actinobacteriota (17.0%). The most abundant genera included *Bacillus* (28.5%), *Methylobacterium-Methylorubrum* (7.0%), *Pseudomonas* (6.6%), etc. (Figure 1a). The 30 day ensiling process effectively enriched Proteobacteria which replaced Firmicutes as the most abundant phylum. The undesired *Enterobacteriaceae* was observed with the highest RA of 35.8%, as well as *Pantoea* (5.3%) at the end. As the major functional LABs, the RA of Lactiplantibacillus and Lacticaseibacillus was only 8.3% at the end. With LP inoculation, the RA of Lactiplantibacillus and Lacticaseibacillus surged and became the dominant genera from day 3 to the end of the ensiling process. Notably, in LPGP, endogenous *Lcb*. *rhamnosus* were the most abundant species of LABs at the mature silage, while LP was only enriched during the first half of the ensiling phase (Figure S2a). As Figure 1b shows, the RA of *unclassified_f__Enterobacteriaceae* (23.37%), *Pediococcus* (22.74%), *Enterobacter* (6.66%), and *Pantoea* (3.17%) in CKGP was significantly lower than that in LPGP (*p* < 0.01), while Lactiplantibacillus (42.2%), Lacticaseibacillus (25.55%), and *Pseudomonas* (1.54%) enriched more in LPGP (*p* < 0.05). *Pseudomonas* enriched more in LPGP (*p* < 0.05) but existed in a low RA for both groups (1.54% in LPGP and 1.34% in CKGP).

**Figure 2:**
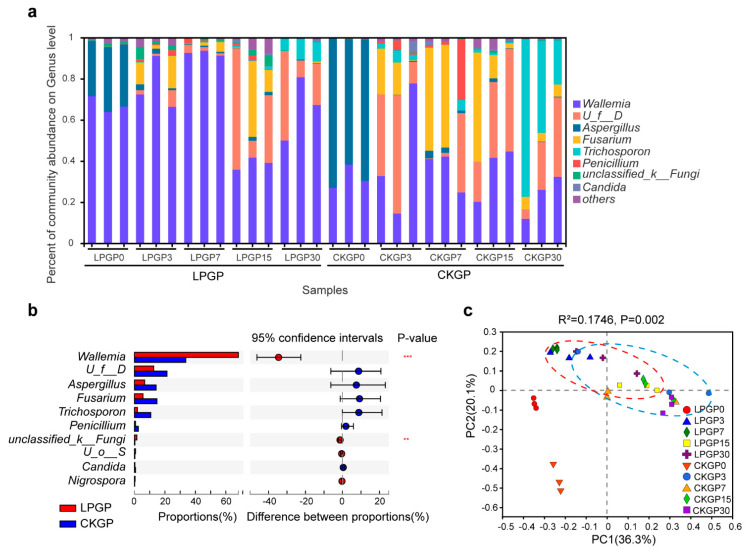
Effect of *Lactiplantibacillus plantarum* additive on the fungal community dynamics of cabbage and rice straw silage. (**a**) The relative abundance of fungal taxa at the genus level; taxa with < 1% of reads were combined as “others”. (**b**) Wilcoxon rank-sum analysis of significant fungal genera between silages without and with inoculation; ***, ** represent *p* < 0.001, *p* < 0.01, respectively. (**c**) Principal coordinate analysis was based on fungal OTU level. CKGP: The control group; LPGP: The inoculation group. The numbers following the CKGP and LPGP indicate the sampling time (day). *U_f__D*, *unclassified_f__Dipodascaceae*; *U_o__S*, *unclassified_o__Saccharomycetes*.

Fermentation time and LP inoculation also markedly influenced the fungal community structure (Figure 2, Figures S1b and S2b, *p* < 0.05). The originally dominant fungal genera were *Wallemia* (in Basidiomycota phylum, 31.9%) and *Aspergillus* (in Ascomycota, 67.4%). At the mature ensiling phase (day 30), the most abundant taxon transitioned to *Trichosporon* (47.6%), followed by *Wallemia* (23.4%), *Dipodascaceae* (20.1%), and *Fusarium* (5.5%). Interestingly, the inoculation of LP remarkably enriched more *Wallemia* (38.9–92.5%) and resulted in fewer total RA of undesirable fungi in this study.

### 3.3. Functional Diversity of Bacterial and Fungal Community

LP inoculation has been found to significantly interfere with microflora succession in silage, and its effects on microflora function diversity were further investigated (Supplementary Materials Spreadsheets 1 and 2). Bacterial phenotypes (oxygen condition, spore-forming, potentially pathogenic, LABs) and fungal functional guilds (soil/undefined saprotroph, animal/plant pathotroph, and other potential pathogenic guilds) were predicted at the species level, respectively. The raw material showed a diverse profile of bacterial phenotypes. In particular, there were abundant obligately aerobic bacteria (such as *Bacillus gibsonii*, *B. marisflavi*, *Pseudomonas parafulva*, *Agrobacterium larrymoorei*, RA 35.4%), spore-forming bacteria (such as *B. gibsonii*, *B. marisflavi*, *Paenibacillus xylanexedens*, RA 27.3%), and potential pathogens (such as *Pseudomonas parafulva*, *unclassified_g__Enterobacter*, RA 14.9%) but rare abundant LABs (such as *Enterococcus faecium*, RA 0.53%) at the initial silage phase (day 0). During the co-ensiling, the aforesaid obligately aerobic and form-spore species in CKGP and LPGP were both significantly inhibited (*p* < 0.05, RA < 1% on day 30). However, CKGP exhibited higher RA of potential pathogens (such as *unclassified_f__Enterobacteriaceae*, *unclassified_g__Enterobacter*, *Weissella cibaria*), especially on days 7 (RA 76.7%) and 30 (RA 50.7%). Epiphytic LABs (such as LP, *Lcb. Rhamnosus*, *Pediococcus pentosaceus*, RA 26.5%) were significantly enriched on day 30. Considering LPGP, there were more LABs (LP, RA 3.5%) than in CKGP at the initial phase (day 0). Upon the completion of the ensiling process, silage with LP inoculation enriched more LABs (such as LP, *Lcb. rhamnosus*, RA 93.7%) and fewer potential pathogens (RA 1.2%).

Regarding the fungal community, the raw material was mainly rich in diverse saprotrophs fungi (such as *unclassified_g__Wallemia*, *Aspergillus penicillioides*, RA 99.6%). However, in CKGP, a few epiphytic animal-pathotroph fungi rapidly accumulated during the ensiling process with RA dynamically changing from 16.7% to 53.4% and kept an RA of 53.4% on day 30 (such as *unclassified_g__Fusarium*, *unclassified_g__Trichosporon*). With the LP inoculation, the RA of animal pathogens was significantly reduced compared to CKGP (2.6–18.1%, *p* < 0.05) and presented an RA of 8.8% at the mature phase. In total, we found LP inoculation significantly declined the animal-pathogenic fungal community during ensiling.

### 3.4. Co-Occurrence Network Analysis for Correlations in Microbial Community

**Figure 3:**
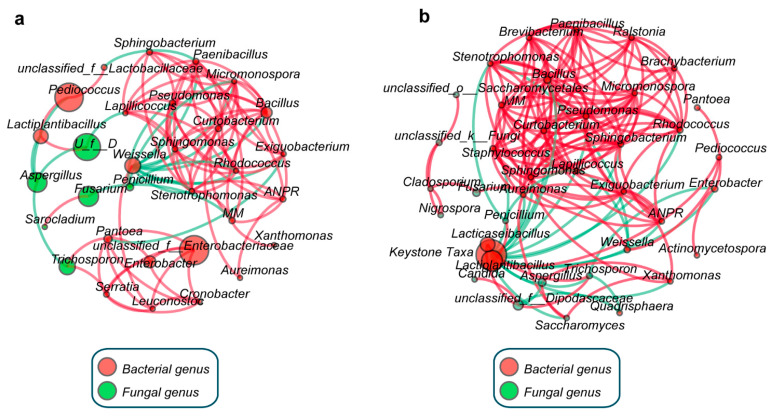
Network analysis showing the co-occurrence based on the correlation among dominant taxa. The control group (**a**); the Lactiplantibacillus *plantarum* inoculation group (**b**). Red edges: Positive correlation; green edges: Negative correlation; the size of nodes indicates the average relative abundance of the genera. *U_f__D*, *unclassified_f__Dipodascaceae*; *MM*, *Methylobacterium-Methylorubrum*; *ANPR*, *Allorhizobium-Neorhizobium-Pararhizobium-Rhizobium*. Keystone taxa include Lactiplantibacillus and *Lacticaseibacillus*.

To better understand how the exogenous LP inoculation interacted with the natural microbial community, co-occurrence networks in two groups were constructed based on the correlations among dominant genera in Figure 3. Notably, the topological characteristics of the co-occurrence network became more complex with the LP inoculation, especially for the average weighted degree (2.996 in CKGP and 6.372 in LPGP, Table S1). Furthermore, the node number, edge number, and diameter of the network increased by 19.4%, 45.0%, and 66.7% in LPGP compared with CKGP. Interestingly, the most abundant genera in CKGP (*Pediococcus*, *unclassified_f__Enterobacteriaceae*, *unclassified_f__Dipodascaceae*, *Fusarium*, *Aspergillus*) had few correlations with others, especially negative correlations (Figure 3a). However, concerning *Weissella* and *Penicillium*, whose average RA (10.63% and 2.6%, respectively) was relatively lower in CKGP, there existed obvious negative correlations (degree 11 and 5, respectively) with other taxa, such as *Pseudomonas*. Interestingly, with LP inoculation, Lactiplantibacillus and *Lacticaseibacillus* turned out to be the most abundant genus (total RA 83.74% from days 3 to 30), which were blinding as keystone taxa with the most negative correlations (degree 12). Furthermore, bacterial genera presented more positive correlations with each other in LPGP compared with CKGP and declined during anaerobic fermentation (Figure 3).

### 3.5. Redundancy Analysis for Correlations of Dominant Microbes and Physicochemical Properties of Silage

**Figure 4:**
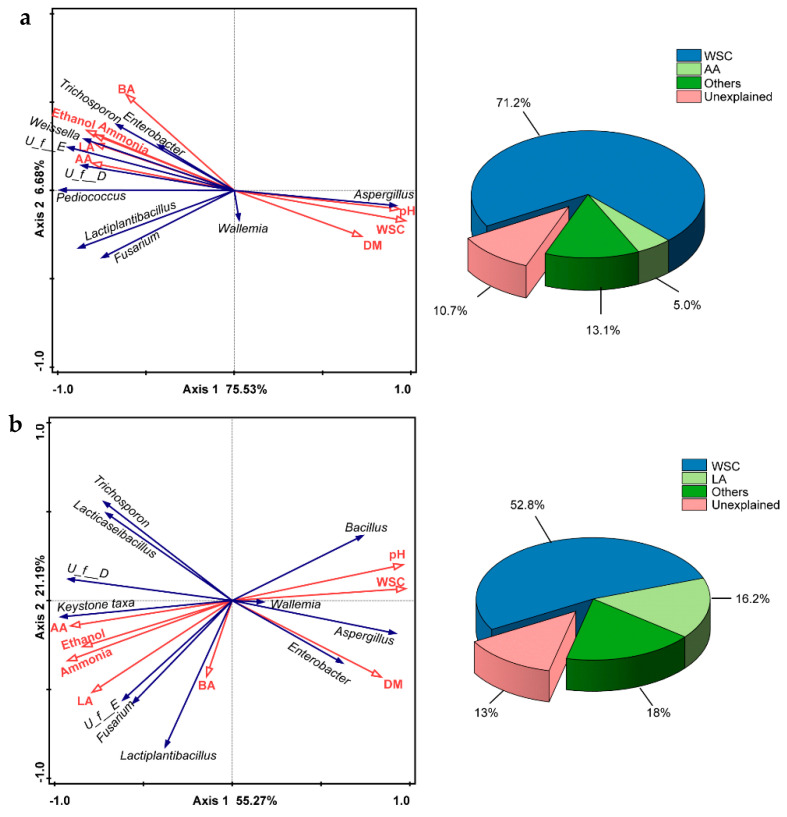
Redundancy analysis (RDA) of relationships between dominant taxa and physicochemical parameters, and explained percentages of variance for each physicochemical parameter. (**a**) Without Lactiplantibacillus *plantarum* inoculation; (**b**) with Lactiplantibacillus *plantarum* inoculation. *U_f__E*, *unclassified_f__Enterobacteriaceae*; *U_f__D*, *unclassified_f__Dipodascaceae*; keystone taxa include Lactiplantibacillus and *Lacticaseibacillus*. DM, dry matter; WSC, water-soluble carbohydrates; LA, lactic acid; AA, acetic acid; BA, butyric acid.

The correlation between dominant taxa and physicochemical properties of silage in CKGP and LPGP was evaluated via redundancy analysis (RDA, Figure 4). The first two axes of RDA accounted for 82.20% and 76.46% of the variance between dominant genera and physicochemical properties in CKGP and LPGP, respectively. WSC (71.2%) and AA (5.0%) were identified to be the key physicochemical properties explaining the succession of dominant microorganisms in CKGP (*p* < 0.05, Figure 4a). In CKGP, WSC was negatively correlated with RA of dominant genera (*Enterobacter, unclassified_f__Enterobacteriaceae*, *Weissella*, *Pediococcus*, *Lactiplantibacillus*, *Trichosporon*, *Fusarium,* and *unclassified_f__Dipodascaceae*) and contents of major microbial metabolites (LA, AA, BA, ethanol, and ammonia), and positively correlated with DM content, pH, and RA of *Aspergillus*. In LPGP, WSC (52.8%) and LA (16.2%) were the key physicochemical properties explaining the microflora succession of silage (*p* < 0.05). WSC showed strong negative correlations with RA of keystone taxa (Lactiplantibacillus and *Lacticaseibacillus*), and contents of microbial metabolites, and was positively correlated with pH, and RA of *Aspergillus*, *Enterobacter*, *Bacillus*.

### 3.6. PLS-PM Analysis to the Effect of Investigated LP Inoculation

**Figure 5:**
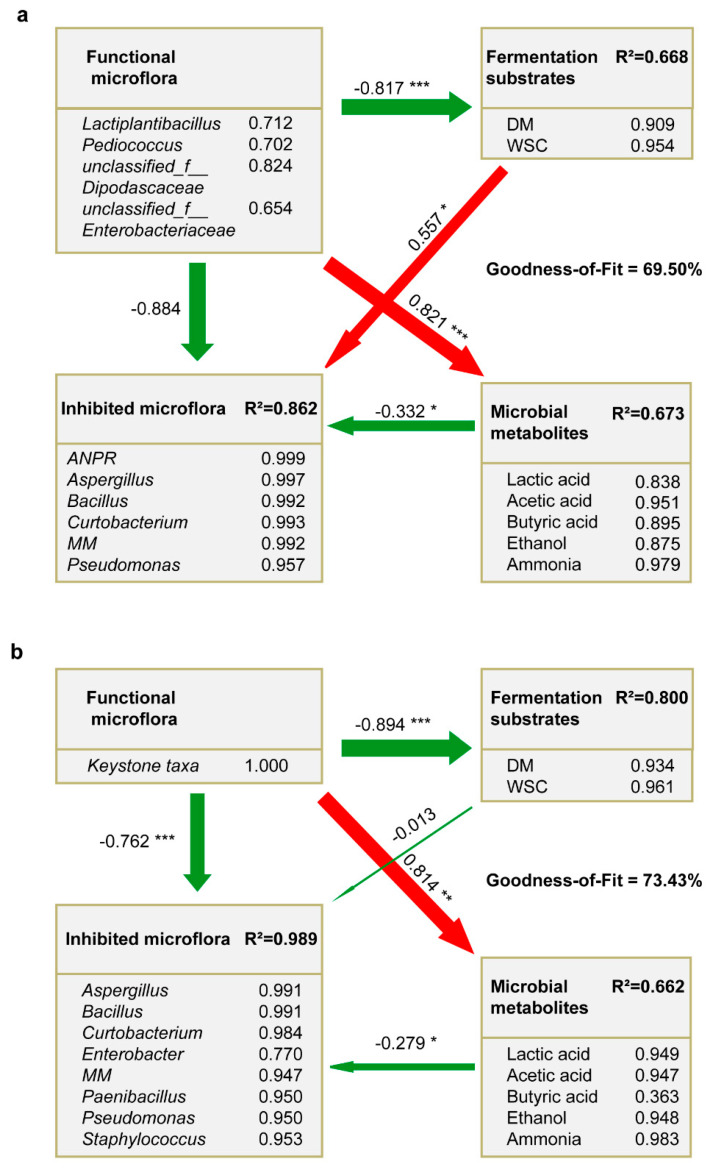
Partial least-squares path model (PLS-PM) showing the effects of functional microbial consortium on inhibited microflora, fermentation characteristics, and microbial metabolite in the control group (**a**) and the inoculation group (**b**). Red arrows indicate the positive correlation while green arrows indicate the negative correlation. Path coefficients and coefficients of determination (R^2^) were calculated after 5000 bootstraps. The width of arrows presents the path coefficients. The numbers and asterisks near arrows indicate path coefficients and *p*-value. ***, **, and * represent *p* < 0.001, *p* < 0.01, and *p* < 0.05, respectively. *ANPR*, *Allorhizobium-Neorhizobium-Pararhizobium-Rhizobium*; *MM*, *Methylobacterium-Methylorubrum*; keystone taxa include Lactiplantibacillus and *Lacticaseibacillus*. DM, dry matter; WSC, water-soluble carbohydrates.

To better understand the augmentation effects from LP inoculation, we further explored the intricate relationships among functional microflora, inhibited/undesirable microflora, fermentation substrates, and microbial metabolites using PLS-PM. Both the direct and indirect effects among different latent variables were evaluated (Figure 5). The goodness of fit over 0.60 indicated good predictive power of these two models, and similar direct effects were found in both models (*p* < 0.05). The functional microflora was negatively related with fermentation substrates (coefficient = −0.817 in CKGP and −0.894 in LPGP, respectively) and was positively correlated with microbial metabolites (coefficient = 0.821 in CKGP and 0.814 in LPGP, respectively), and microbial metabolites were negatively related with inhibited microflora (coefficient = −0.332 in CKGP and −0.279 in LPGP, respectively).

In CKGP, functional microflora was observed to be indirectly correlated with undesirable microflora by fermentation substrates (coefficient = −0.455, *p* = 0.002) and microbial metabolites (coefficient = −0.273, *p* = 0.033, Figure 5a). However, the direct correlation between functional microflora and inhibited microflora was not significant (coefficient = −0.884, *p* = 0.387). On the contrary, in LPGP, keystone taxa (Lactiplantibacillus and *Lacticaseibacillus*) became the main functional microorganism (Figure 1a) and negatively correlated with inhibited microflora in a strongly direct way (coefficient = 0.762, *p* < 0.001; Figure 5b). However, the indirect correlations with inhibited microflora by fermentation substrate (coefficient = 0.012, *p* = 0.923) and microbial metabolites (coefficient = −0.227, *p* = 0.063, Figure 5b) were not significant.

## 4. Discussion

Vegetable waste, such as cauliflower and cabbage leaf byproducts, are ubiquitous in landfills but rarely reported to be bio-transformed as animal feed, such as silage [2]. LP could be an efficient microbial inoculation to enhance the fermentation quality of ensiling different materials [2,9,11,25,26] but the underlying effect and intricate relationships have been little investigated. This present study disposed of cabbage byproducts as added-value silage and was the first to focus on the underlying effect of LP improving silage quality and microbial community functional diversity in food microbial areas.

### 4.1. The Effects of LP Inoculation on Physicochemical Properties of Silage

The minimum DM (>200 g·kg^−1^ FM) and WSC content (>50 g·kg^−1^ DM) were reported necessary as LABs transformed the WSC to organic acid, e.g., LA, to decrease the pH and preserve the forage successfully [9,27]. Thus, the formula of initial materials was suitable in this study. During the ensiling process, the major functional microorganisms such as LABs and yeasts would degrade organic matters, especially those readily degradable ones (e.g., WSC), to organic acids and ethanol [2,6]. Hence, the recalcitrant lignocellulosic components accumulated while the WSC, DM, CF content decreased after fermentation (Table 2 and Table 3). The LP inoculation significantly increased the DM content of silage samples, which indicated a better performance on conservation and valorization of organic matters (Table 2). The higher DM content preserved in LPGP could be explained by the LP inoculation increasing the LA content, which could decrease pH and resist undesirable and fast-metabolic microbiome, such as pathogens and deterioration [6]. The LP inoculation also resulted in a higher content of LA and lower content of AA and BA in LPGP, due to the enhanced homolactic fermentation. Consequently, more organic matters would be preserved, which was consistent with increasing DM content in this study [2,9]. Flieg’s score based on the organic acid ingredient was reported as the important evaluation proxy for silage quality, which could indicate the odor characteristic in a way [4,28]. In this study, the LP inoculation remarkably increased Flieg’s score (Table 4), and the silage quality was evaluated as “very good”. Hence, inoculation of exogenous LP could be a feasible method to transform cabbage waste and rice straw into animal feed. The inoculation significantly reduced ammonia-N content, which could be explained by the fact that more LABs and LA could effectively restrain undesirable bacteria such as Enterobacteria from degrading protein and oligopeptide to ammonia [6,9]. Moreover, ammonia-N below 7% and beyond 10% indicated a successful silage fermentation and severe nutritional loss, respectively [29]. The LP inoculation effectively inhibited the nutritional loss during the silage.

Moreover, the LP inoculation did not facilitate the accumulation of CF and CP, while it basically maintained the value of these two important nutrition indicators (Table 3). The ADF and NDF content declined during the silage; in particular, the ADF content significantly declined at the end of co-ensiling (*p* < 0.05), which was different from Mu et al.’s report that LP played no significant role in ADF and NDF content in amaranth and rice straw silage [9]. This different result could account for more organic acid to accelerate the lignocellulose hydrolysis and the different raw materials in the silage study [27].

### 4.2. Dynamic Variations of Microbial Composition and Functional Diversity

The specific rDNA sequencing method was widely applied to investigate microbial community succession in silage [25]. The coverage index of all samples was almost 1.0 indicating the DNA sequencing results were representative of the microbial communities (Table 5). According to the declining Shannon and increasing Simpson indexes of bacterial community in LPGP, LP inoculation significantly decreased the diversity of bacterial communities during the fermentation (*p* < 0.05), while it played an insignificant role in bacterial community richness and fungal community diversity and richness. These results could be explained by the fact that exogenous LP inoculation resisted the undesirable bacteria and interfered with bacterial community diversity, which was consistent with what Keshri et al. reported in wheat silage [25]. As Figure 1a shows, undesirable microorganisms were enriched in CKGP, such as Enterobacteriaceae (*unclassified_f__Enterobacteriaceae*, *Enterobacter*) and *Pantoea*. Both of them were reported for their proteolytic activity, which could lead to inefficient LA productivity, higher pH, and higher ammonia content [6,30]. Generally, inadequate epiphytic LABs (<10^5^ CFU·g^−1^ FM) and abundant aerobic bacteria (> 10^6^ CFU·g^−1^ FM) could result in low-quality silage due to the inefficient lactic fermentation and aerobic deterioration during ensiling [2,6], which was consistent with our results in CKGP. With the LP inoculation, *Lactiplantibacillus* and *Lacticaseibacillus* became the most abundant genus and LABs (total RA 83.74% from days 3 to 30), which was also frequently found and provided a stable fermentative environment in other silage [6]. Interestingly, the main LABs shifted from LP during the first 15 days to *Lcb. rhamnosus* during the last 15 days (Figure S2a). Therefore, LP and *Lcb. rhamnosus* could play an important role in the first and late half phase, respectively, during co-ensiling of cabbage byproduct and rice straw. Furthermore, LP and *Lcb. rhamnosus* were facultative anaerobic and fastidious anaerobic LABs, respectively, so the available oxygen content in the first 15 days could be more suitable for facultative anaerobic LP. *Lcb. rhamnosus* was enriched more in the last 15 days, which might result from less O_2_ and more CO_2_ [31]_._ To consider the blinding effect of these two taxa, we assumed that *Lactiplantibacillus* and *Lacticaseibacillus* were the keystone taxa in LPGP and then focused on the effect of keystone taxa on improving silage quality. *Pseudomonas* was more abundant in LPGP and was also regarded as undesirable bacteria due to its ability to produce biogenic amines [10]. However, its RA was relatively low in both groups. *Pediococcus* was the most dominant LAB in CKGP. Comparatively, *Lactiplantibacillus* and *Lacticaseibacillus* became the most dominant taxa with the LP inoculation. Moreover, *Lactiplantibacillus* and *Lacticaseibacillus* were reported as more tolerant of low pH and more effective homofermentative LABs than *Pediococcus* during co-ensiling of amaranth and rice straw [9], which was consistent with our results. Comparatively, the exogenous LP inoculation could significantly improve the RA of homofermentative and efficient LABs in silage while decreasing the undesirable bacteria (*p* < 0.05).

Based on previous reports, *Trichosporon* and *Fusarium* are pathogenic fungi of humans and crops and should be prevented in final silage products [32,33]. However, they were enriched dominantly in CKGP. LP inoculation remarkably enriched more *Wallemia*, which were reported as saprotroph fungi and resulted in lower total RA of undesirable fungi in this study, such as *Aspergillus, Trichosporon*, and *Fusarium* [10,34,35]. Therefore, LP inoculation could inhibit these mold and pathogenic fungi, which could reduce the accumulation of mycotoxins and improve the safety quality of silage [36]. However, limited researchers noticed the role of LP inoculation in the fungal pathogens in silage production.

In the present study, the epiphytic microbiome including obligately aerobic, spore-forming, and potentially pathogenic bacteria, and a few animal-pathotroph fungi were rapidly enriched in CKGP during the ensiling process. As previously reported, aerobic microbes could result in aerobic deterioration and poor nutrition preservation. They hardly survived in an anaerobic environment, and usually showed a declining RA along the ensiling process [6,17]. The spore-forming *Bacillus* in this study was facultative anaerobic and could tolerate low pH and the anaerobic environment to some extent. Their spores could exist in silage and ruminant intestinal tract, which were closely associated with aerobic silage deterioration and spore contamination in livestock products (e.g., milk) [6]. Furthermore, epiphytic animal-pathotroph fungi, such as *Trichosporon* and *Fusarium*, and Gram-negative *Enterobacter*, *unclassified_f__Enterobacteriaceae* in CKGP, usually produced multiple mycotoxins and endotoxin when present in animal feeds; these toxins could result in poor-quality dairy cow performance and endanger both animal and human health [6,7]. Therefore, the bacterial species characterized as aerobic/spore-forming/potentially pathogenic and animal-pathotroph fungi are detrimental in silage production. With the LP inoculation, these aforementioned undesirable microorganisms were significantly resisted. However, there are few studies that systematically focus on microbial functional diversity related to silage production.

### 4.3. Correlations of Dominant Microbes and Physicochemical Properties of Silage

As Banerjee et al. reported, negative correlations of co-occurrence networks indicated possible competition for resources and growth inhibition, while positive indicated common predators within microbial taxa [14]. Interestingly, the most abundant genera in CKGP (*Pediococcus*, *unclassified_f__Enterobacteriaceae*, *Fusarium*, *Aspergillus*) had few correlations with others, especially negative correlations (Figure 3a). However, *Weissella* and *Penicillium* whose average RA (10.63% and 2.6%, respectively) was relatively lower were identified as the keystone taxa in CKGP due to their obvious negative correlations (degree 11 and 5, respectively) with inhibited taxa, such as *Pseudomonas*. Interestingly, with LP inoculation, keystone taxa (*Lactiplantibacillus* and *Lacticaseibacillus*) turned out to be the most abundant genus (RA 67.8%). Based on that, it was plausible that the keystone taxa in CKGP with low RA were not sufficient to restrain the undesirable microbe. On the contrary, LP inoculation significantly increased the RA of keystone taxa (*Lactiplantibacillus* and *Lacticaseibacillus*) and improved the overall functionality of the microbial community in LPGP.

Correlations among dominant taxa and physicochemical indexes in CKGP and LPGP were similar (Figure 4). DM, pH, and WSC content were negatively related to organic acids, ethanol, and ammonia content as well as some dominant taxa. These correlations indicated the aforementioned dominant genera could metabolize WSC and other organic components to organic acids, ammonia, and ethanol, therefore consuming DM, reducing pH, and inhibiting undesirable taxa, such as *Aspergillus* in CKGP and *Aspergillus*, *Bacillus*, and *Enterobacter* in LPGP. However, dominant taxa and the correlations among dominant taxa changed obviously with the inoculation of LP (Figure 4b). These results could be explained by the fact that exogenous LP inoculation increased the RA of keystone taxa (*Lactiplantibacillus* and *Lacticaseibacillus*), which could provide efficient homofermentation and resist undesired taxa (*Aspergillus*, *Enterobacter*, *Bacillus*). These results were consistent with previous studies that LP inoculation increased the RA of *Lactiplantibacillus* in silage, improved silage microbial community and fermentation metabolites [2,9].

### 4.4. Intricate Relationships of LP Inoculation Augmentation Effects

With LP inoculation, more keystone taxa (*Lactiplantibacillus* and *Lacticaseibacillus*) were enriched, which effectively inhibited undesirable microflora and improved the fermentation quality of silage. As PLS-PM analysis revealed (Figure 5), the similar relationships between CKGP and LPGP indicated that functional microflora could utilize fermentation substrate and facilitate the production of organic acids, ammonia, and ethanol. Meanwhile, these metabolites could resist some undesirable microflora, such as *Aspergillus* and *Bacillus*. These results were consistent with the discussion about correlations between dominant taxa and physicochemical indexes in Section 4.3.

Notably, functional microflora in CKGP indirectly resisted undesirable microflora by fermentation substrate competition and microbial metabolite inhibition rather than direct interactions between two types of microorganisms. Comparatively, functional microflora (keystone taxa: *Lactiplantibacillus* and *Lacticaseibacillus*) in silage inoculated with LP primarily worked through directly inhibiting undesirable microflora, rather than indirect effects including competition for fermentation substrate and inhibition by producing microbial metabolites. These hypotheses were different from the conventional perspective that LABs transform WSC into organic acids to reduce pH and inhibit the undesirable microorganisms [2,6,9], and are reasonably based on the current analysis results.

## 5. Conclusions

In co-ensiling of cabbage byproduct and rice straw, the inoculation of exogenous LP significantly improved the ensiling quality indicated by the elevated contents of DM and LA, and reduced the contents of ammonia, AA, BA, ADF, and the pH value, which was closely associated with the effective homolactic fermentation system. Specifically, LP inoculation significantly influenced the structure of the microbial community, improved the proportion of functional types needed by successful ensiling, and resisted the undesired microorganisms, especially aerobic, spore-forming, and pathogenic taxa. With LP inoculation, *Lactiplantibacillus* and *Lacticaseibacillus* were observed as the keystone taxa and major functional species, which directly inhibited undesirable microbes and improved the fermentation characteristics.

## Figures and Tables

**Table 1:** The compositions of the raw materials.

Material	Rice Straw	Cabbage Leaf	Corn Flour
DM (Dry matter, g·kg^−1^)	864.7 ± 1.3	59.4 ± 0.7	877.3 ± 1.9
WSC (Water-soluble carbohydrates, g·kg^−1^ DM)	29.5 ± 1.7	439.4 ± 36.4	71.8 ± 2.9
CF (Crude fat, g·kg^−1^ DM)	98.9 ± 6.1	425.0 ± 2.6	52.9 ± 3.5
CP (Crude Protein, g·kg^−1^ DM)	76.3 ± 1.9	220.0 ± 5.7	90.5 ± 6.1
ADF (Acid detergent fiber, g·kg^−1^ DM)	428.9 ± 16.1	176.9 ± 7.8	11.8 ± 1.3
NDF (Neutral detergent fiber, g·kg^−1^ DM)	676.6 ± 13.0	233.6 ± 4.8	24.7 ± 2.0

The values are shown as the mean ± standard deviation of three replicates.

**Table 2:** Effects of inoculation and fermentation time on fermented characteristics of the silage.

Item	Time (Days)					SEM	Significance		
	0	3	7	15	30		TR	TI	TR × TI
DM (Dry matter, g·kg^−1^)									
CKGP	309.5 ± 6.1 ^Aa^	295.9 ± 3.4 ^Ab^	285.3 ± 0.7 ^Bb^	287.0 ± 5.7 ^Ab^	270.9 ± 5.5 ^Bc^	2.255	***	***	0.268
LPGP	310.3 ± 4.5 ^Aa^	299.7 ± 9.2 ^Aab^	292.1 ± 4.1 ^Abc^	291.8 ± 4.1 ^Abc^	283.4 ± 2.5 ^Ac^				
pH									
CKGP	5.93 ± 0.03 ^Aa^	4.48 ± 0.09 ^Ab^	4.24 ± 0.08 ^Ac^	3.79 ± 0.06 ^Ae^	3.99 ± 0.07 ^Ad^	0.157	***	***	***
LPGP	5.87 ± 0.06 ^Aa^	3.78 ± 0.04 ^Bb^	3.55 ± 0.06^Bc^	3.55 ± 0.09 ^Bc^	3.80 ± 0.11 ^Ab^				
WSC (g·kg^−1^ DM)									
CKGP	68.8 ± 5.1 ^Aa^	13.9 ± 1.3 ^Bb^	20.2 ± 7.8 ^Ab^	13.8 ± 5.3 ^Ab^	12.5 ± 5.7 ^Ab^	4.214	0.306	***	*
LPGP	68.7 ± 5.9 ^Aa^	23.3 ± 0.2 ^Ab^	7.4 ± 0.9 ^Ac^	9.8 ± 1.7 ^Ac^	10.8 ± 0.1 ^Ac^				
Lactic acid (g·kg^−1^ DM)									
CKGP	0.0 ± 0.0 ^Ac^	21.3 ± 3.4 ^Bb^	23.7 ± 1.3 ^Ba^	35.8 ± 4.5 ^Bb^	22.4 ± 1.4 ^Ab^	3.279	***	***	***
LPGP	0.0 ± 0.0 ^Ad^	38.3 ± 1.2 ^Ab^	46.6 ± 3 ^Aab^	52.1 ± 7 ^Aa^	25.3 ± 0.8 ^Ac^				
Acetic acid (g·kg^−1^ DM)									
CKGP	0.0 ± 0.0 ^Ad^	12.8 ± 0.8 ^Aa^	7.7 ± 0.6 ^Ab^	8.3 ± 1.7 ^Abc^	5.4 ± 0.4 ^Ac^	0.720	***	***	***
LPGP	0.0 ± 0.0 ^Ad^	4.6 ± 0.4 ^Bb^	5.0 ± 0.1 ^Bb^	6.0 ± 0.3 ^Aa^	3.7 ± 0.2 ^Ac^				
Butyric acid (g·kg^−1^ DM)									
CKGP	0.0 ± 0.0 ^Ab^	5.0 ± 1.6 ^Aa^	2.5 ± 0.1 ^Aa^	2.5 ± 0.9 ^Aab^	2.4 ± 0.5 ^Aab^	0.329	***	***	***
LPGP	0.0 ± 0.0 ^Ab^	0.3 ± 0.0 ^Ba^	0.3 ± 0.0 ^Bab^	0.1 ± 0.2 ^Bab^	0.0 ± 0.0 ^Bb^				
Ethanol (g·kg^−1^ DM)									
CKGP	0.0 ± 0.0 ^Ab^	9.9 ± 0.7 ^Aa^	6.7 ± 1.1 ^Ba^	7.1 ± 1.8 ^Aa^	6.6 ± 0.8 ^Aa^	0.828	0.274	***	0.051
LPGP	0.0 ± 0.0 ^Ab^	7.3 ± 0.1 ^Ba^	12.5 ± 2 ^Aa^	11.5 ± 4.1 ^Aa^	7.9 ± 1.7 ^Aa^				
Ammonia/total N (%)									
CKGP	1.1 ± 0.1 ^Ac^	11.9 ± 0.3 ^Aa^	8.6 ± 0. 1^Aa^	8.6 ± 1.2 ^Aab^	6.4 ± 0.6 ^Ab^	0.644	***	***	***
LPGP	0.9 ± 0.1 ^Bc^	4.5 ± 0.4 ^Bab^	5.1 ± 0.3 ^Ba^	5.7 ± 0.5 ^Ba^	4.1 ± 0.2 ^Bb^				

WSC: Water-soluble carbohydrates; CKGP: The control group; LPGP: The inoculated group. Capital letters indicate the significant difference between the control and inoculated groups on the same sampling day (*p* < 0.05). Lowercase letters indicate the significant differences between the different fermented times (*p* < 0.05). The values are shown as the mean ± standard deviation of three replicates. SEM = standard error of means. TR: Treatment; TI: Time; TR × TI: The interaction between treatment and temperature; *: *p* < 0.05; ***: *p* < 0.001.

**Table 3:** Effects of inoculation and fermented time on nutrition composition of the silage.

Item	Time (Days)					SEM	Significance		
	0	3	7	15	30		TR	TI	TR × TI
CF (Crude fat, g × kg^−1^ DM)									
CKGP	145.9 ± 8.6 ^Aa^	112.7 ± 5.1 ^Ab^	142.9 ± 4.6 ^Aa^	150.1 ± 3.0 ^Aa^	121.1 ± 3.2 ^Ab^	2.916	0.458	***	0.372
LPGP	142.4 ± 8.8 ^Aa^	125.9 ± 16.3 ^Aab^	129.7 ± 1.7 ^Bab^	151 ± 8.5 ^Aa^	111.6 ± 8.4 ^Ab^				
CP (Crude Protein, g × kg^−1^ DM)									
CKGP	99.7 ± 1.2 ^Aa^	101 ± 3.5 ^Aa^	102.4 ± 1.7 ^Aa^	102.1 ± 3.3 ^Aa^	102.3 ± 2.2 ^Aa^	0.476	0.379	0.133	0.275
LPGP	98.8 ± 0.7 ^Aa^	102.5 ± 1.6 ^Aa^	99.1 ± 2.3 ^Aa^	104.4 ± 2.1 ^Aa^	101.6 ± 3.4 ^Aa^				
Acid detergent fiber (ADF, g × kg^−1^ DM)									
CKGP	202.4 ± 2.3 ^Ac^	233.0 ± 1.2 ^Ab^	230.6 ± 3.1 ^Ab^	248.7 ± 7.3 ^Aa^	238.4 ± 3.8 ^Aab^	2.870	***	**	***
LPGP	215.6 ± 6.4 ^Aab^	211.8 ± 3.5 ^Bbc^	224.8 ± 4.6 ^Aab^	199.4 ± 1.7 ^Bc^	227.8 ± 1.5 ^Ba^				
Neutral detergent fiber (NDF, g × kg^−1^ DM)									
CKGP	301.5 ± 3.2 ^Ac^	343.6 ± 8.8 ^Aa^	322.3 ± 5.7 ^Ab^	324.2 ± 1.5 ^Ab^	316.9 ± 5.0 ^Aab^	2.996	*	***	***
LPGP	302.6 ± 4.3 ^Ac^	304.6 ± 6.8 ^Bbc^	325.4 ± 5.7 ^Aab^	306.4 ± 0.3 ^Bbc^	335.9 ± 13.4 ^Aa^				

DM: Dry matter. CKGP: The control group; LPGP: The inoculated group. Capital letters indicate the significant difference between the control and inoculated groups on the same sampling day (*p* < 0.05). Lowercase letters indicate the significant differences between the different fermented times (*p* < 0.05). The values are shown as the mean ± standard deviation of three replicates. SEM = standard error of means. TR: Treatment; TI: Time; TR × TI: The interaction between treatment and temperature; *: *p* < 0.05; **: *p* < 0.01; ***: *p* < 0.001.

**Table 4:** Ensiling quality evaluated by Flieg’s score based on the concentration of organic acid.

Item	Time (Days)					SEM	Significance				
	0	3	7	15	30		TR	TI		TR × TI	
Formic acid (g·kg^−1^ DM)											
CKGP	0.0 ± 0.0 ^Ab^	5.61 ± 0.76 ^Ba^	8.24 ± 1.13 ^Ba^	8.76 ± 0.54 ^Aa^	6.82 ± 2.58 ^Aa^	0.296	***		***		***
LPGP	0.0 ± 0.0 ^Ad^	12.21 ± 0.92 ^Ab^	13.19±0.33 ^Ab^	16.66 ± 1.90 ^Aa^	4.44 ± 0.89 ^Ac^	-					
Propionic acid (g·kg^−1^ DM)											
CKGP	0.0 ± 0.0 ^Ad^	0.64 ± 0.02 ^Bc^	1.03 ± 0.08 ^Bb^	1.27 ± 0.0 ^Ba^	1.28 ± 0.0 ^Ba^	0.059	***		***		***
LPGP	0.0 ± 0.0 ^Ad^	0.90 ± 0.06 ^Ac^	1.87 ± 0.03 ^Ab^	3.09 ± 0.51 ^Ba^	2.56 ± 0.10 ^Aab^	-					
Lactic acid/TOA (%)											
CKGP	NA	52.06	63.27	72.47	69.15	-	-				
LPGP	NA	81.31	83.36	81.41	80.76	-					
Acetic acid/TOA (%)											
CKGP	NA	31.32	23.10	16.72	16.77	-	-				
LPGP	NA	9.42	8.61	9.43	12.47	-					
Butyric acid/TOA (%)											
CKGP	NA	12.12	6.55	4.99	7.31	-	-				
LPGP	NA	0.92	0.51	0.16	0.00	-					
Flieg’s evalution (score)											
CKGP	NA	Bad (31)	Medium (45)	Good (61)	Medium (53)	-	-				
LPGP	NA	Very good (100)	Very good (100)	Very good (100)	Very good (100)	-					

DM: Dry matter; TOA: Total organic acid including the content of lactic acid, formic acid, acetic acid, propionic acid, butyric acid. CKGP: The control group; LPGP: The inoculated group. Capital letters indicate the significant difference between the control and inoculated groups on the same sampling day (*p* < 0.05). Lowercase letters indicate the significant differences between the different fermented times (*p* < 0.05). The values are shown as the mean ± standard deviation of three replicates. SEM = standard error of means. NA: Not calculated; TR: Treatment; TI: Time; TR × TI: The interaction between treatment and temperature; *** *p* < 0.001.

**Table 5:** Alpha diversity of bacterial and fungal community during the ensiling.

Community	Treatment	Time (Days)	Coverage	Chao1	Shannon	Simpson	Ace
Bacterial community	LPGP	0	1.00 ± 0.00	222.3 ± 0.93	3.79 ± 0.07	0.04 ± 0.00	222.37 ± 1.87
		3	1.00 ± 0.00	106.1 ± 32.3	0.74 ± 0.14	0.73 ± 0.06	129.15 ± 43.81
		7	1.00 ± 0.00	147.4 ± 10.0	0.90 ± 0.17	0.68 ± 0.07	168.09 ± 20.69
		15	1.00 ± 0.00	190.4 ± 12.4	1.93 ± 0.30	0.27 ± 0.08	187.06 ± 14.56
		30	1.00 ± 0.00	158.0 ± 10.7	0.56 ± 0.18	0.84 ± 0.05	154.51 ± 20.81
	CKGP	0	1.00 ± 0.00	218.4 ± 6.37	3.66 ± 0.06	0.06 ± 0.01	217.31 ± 6.69
		3	1.00 ± 0.00	117.8 ± 26.8	1.82 ± 0.16	0.28 ± 0.05	130.21 ± 41.01
		7	1.00 ± 0.00	119.8 ± 45.5	1.76 ± 0.14	0.28 ± 0.05	142.55 ± 58.92
		15	1.00 ± 0.00	176.2 ± 51.6	1.89 ± 0.15	0.26 ± 0.06	191.51 ± 50.8
		30	1.00 ± 0.00	174.0 ± 10.9	2.24 ± 0.08	0.21 ± 0.03	172.22 ± 6.22
Fungal community	LPGP	0	1.00 ± 0.00	34.4 ± 2.51	1.99 ± 0.0	0.19 ± 0.02	222.37 ± 1.87
		3	1.00 ± 0.00	36.7 ± 1.04	1.92 ± 0.31	0.23 ± 0.08	129.15 ± 43.81
		7	1.00 ± 0.00	30.1 ± 2.71	1.52 ± 0.05	0.30 ± 0.02	168.09 ± 20.69
		15	1.00 ± 0.00	33.4 ± 7.06	2.18 ± 0.29	0.17 ± 0.05	187.06 ± 14.56
		30	1.00 ± 0.00	31.0 ± 5.51	1.90 ± 0.14	0.20 ± 0.04	154.51 ± 20.81
	CKGP	0	1.00 ± 0.00	29.6 ± 4.03	1.83 ± 0.07	0.23 ± 0.03	217.31 ± 6.69
		3	1.00 ± 0.00	37.8 ± 14.8	2.00 ± 0.04	0.18 ± 0.02	130.21 ± 41.01
		7	1.00 ± 0.00	35.6 ± 4.80	1.75 ± 0.21	0.27 ± 0.08	142.55 ± 58.92
		15	1.00 ± 0.00	33.6 ± 7.88	2.02 ± 0.31	0.21 ± 0.10	191.51 ± 50.8
		30	1.00 ± 0.00	32.6 ± 7.94	1.66 ± 0.57	0.33 ± 0.24	172.22 ± 6.22

LPGP: The inoculation group; CKGP: The control group. The number followed LPGP/CKGP indicates sampling time (day). The mean ± standard deviation of three replicates.

## Data Availability

The datasets generated for this study can be found in Sequence Read Archive under BioProject, PRJNA686717 (https://www.ncbi.nlm.nih.gov/sra/, accessed on 20 December 2020).

## Supplementary Materials

The following are available online at https://www.mdpi.com/article/10.3390/microorganisms9051099/s1, Figure S1: Effect of *Lactiplantibacillus plantarum* additive on the microbial community dynamics at the phylum level of cabbage and rice straw silage. Figure S2: Effect of *Lactiplantibacillus plantarum* additive on the microbial community dynamics at the species level of cabbage and rice straw silage. Table S1: Topological characteristics of co-occurence network., Spreadsheet S1: The bacterial phenotypes annotation using DacDive database and Bergey’s Manual of Systematic Bacteriology., Spreadsheet S2: The fungal trophic modes and ecological guilds annotation using FUNGuild.
