# Supplementary material for: Keystone Taxa Lactiplantibacillus and Lacticaseibacillus Directly Improve the Ensiling Performance and Microflora Profile in Co-Ensiling Cabbage Byproduct and Rice Straw
Source: Microorganisms. 2021 May 20;9(5):1099. doi: 10.3390/microorganisms9051099 (PMC8161039; doi:10.3390/microorganisms9051099)
Supplement: Supplementary file 1 [file microorganisms-09-01099-s001.zip › Supplementary_Material.pdf]

Supplementary data list

Spreadsheet 1 The bacterial phenotypes annotation using DacDive database and Bergey' s Manual of Systematic Bacteriology. Only taxa of RA > 5% was annotated.

Spreadsheet 2 The fungal trophic modes and ecological guilds annotation using FUNGuild.

Supplementary Figure S1. Effect of *Lactobacillus plantarum* additive on the microbial community dynamics at the phylum level of cabbage and rice straw silage. (A) The relative abundance of bacterial taxa; (B) The relative abundance of fungal taxa; Taxa with < 5% of reads were combined as '*others*'; CKGP: the control group; LPGP: the inoculation group. The numbers following the CKGP and LPGP indicate the sampling time (day).

Supplementary Figure S2. Effect of *Lactobacillus plantarum* additive on the microbial community dynamics at the species level of cabbage and rice straw silage. (A) The relative abundance of bacterial taxa; (B) The relative abundance of fungal taxa; Taxa with < 5% of reads were combined as 'others'; CKGP: the control group; LPGP: the inoculation group. The numbers following the CKGP and LPGP indicate the sampling time (day).

Supplementary Table S1 Topological characteristics of co-occurence network.

1. Supplementary Figures

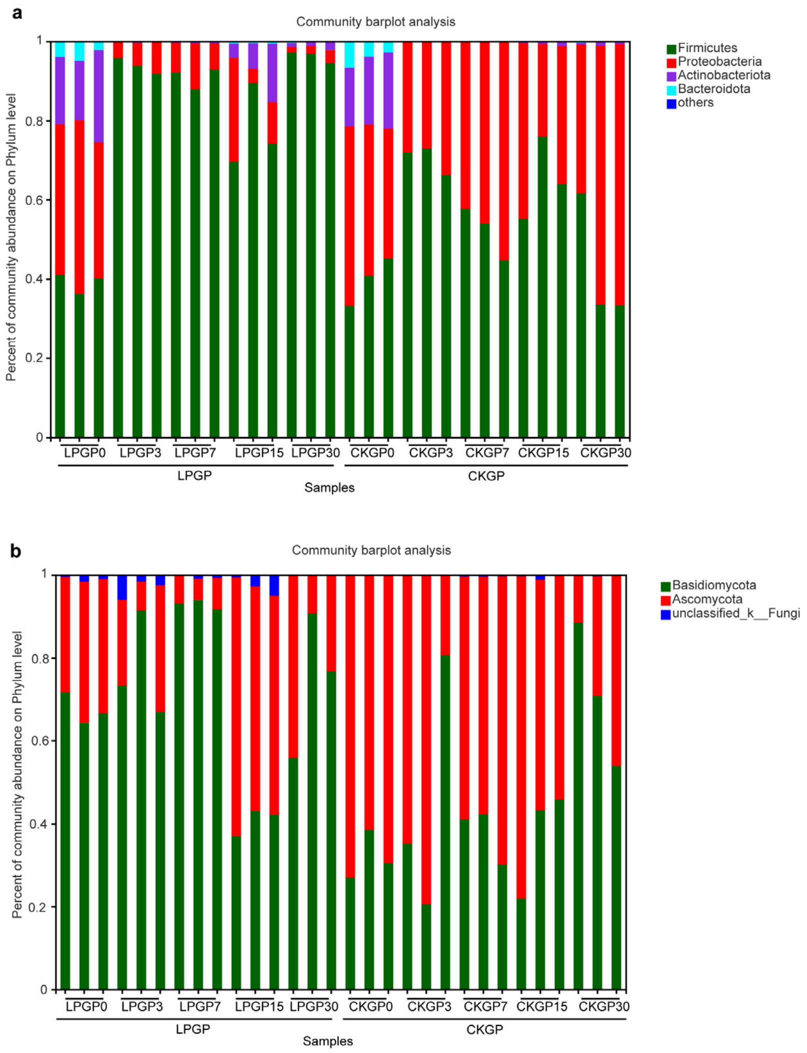

Supplementary Figure S1. Effect of *Lactobacillus plantarum* additive on the microbial community dynamics at the phylum level of cabbage and rice straw silage. (A) The relative abundance of bacterial taxa; (B) The relative abundance of fungal taxa; Taxa with < 5% of reads were combined as '*others*'; CKGP: the control group; LPGP: the inoculation group. The numbers following the CKGP and LPGP indicate the sampling time (day).

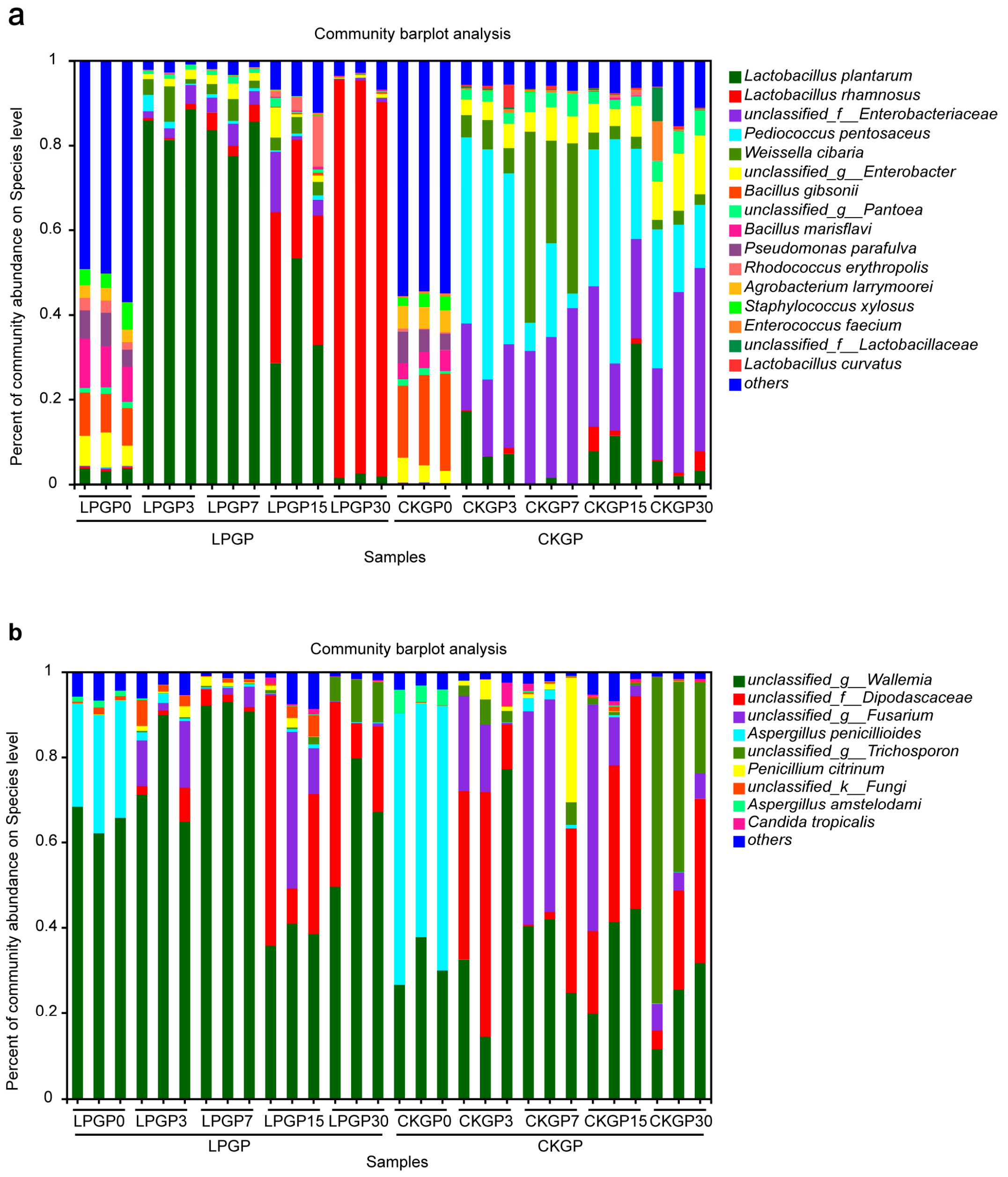

Supplementary Figure S2. Effect of *Lactobacillus plantarum* additive on the microbial community dynamics at the species level of cabbage and rice straw silage. (A) The relative abundance of bacterial taxa; (B) The relative abundance of fungal taxa; Taxa with < 5% of reads were combined as 'others'; CKGP: the control group; LPGP: the inoculation group. The numbers following the CKGP and LPGP indicate the sampling time (day).

2. Supplementary Tables

Supplementary Table S1 Topological characteristics of co-occurence network.

Parameter	Group	
	CKGP	LPGP
Total nodes	31	37
Total edges	100	145
Average degree	6.452	7.838
Average weighted degree	2.996	6.372
Network diameter	6	10
Graph density	0.215	0.218
Modularity	0.649	0.312
Connected components	1	1
Clustering coefficient	0.621	0.632
Average path length	2.634	3.239

CKGP: the control group; LPGP: the inoculation group.
